# Mass spectrometric analysis of digesta does not improve the allergenicity assessment of GM crops

**DOI:** 10.1007/s11248-021-00254-x

**Published:** 2021-04-16

**Authors:** Rod A. Herman, Patricia A. Bauman, Laurie Goodwin, Emir Islamovic, Eric H. Ma, Hector Serrano, Andre Silvanovich, Abigail R. Simmons, Ping Song, Afua O. Tetteh, Rong Wang

**Affiliations:** 1 Corteva Agriscience, Indianapolis, IN USA; 2 Syngenta Crop Protection, LLC., Research Triangle Park, NC USA; 3 CropLife International, Arlington, VA USA; 4 BASF Corporation, Research Triangle Park, NC USA; 5 Bayer, Crop Science Division, Chesterfield, MO USA

**Keywords:** Plant biotechnology, Allergen, Mass spectrometry, Digestibility

## Abstract

An investigation of the potential allergenicity of newly expressed proteins in genetically modified (GM) crops comprises part of the assessment of GM crop safety. However, allergenicity is not completely predictable from a definitive assay result or set of protein characteristics, and scientific opinions regarding the data that should be used to assess allergenicity are continuously evolving. Early studies supported a correlation between the stability of a protein exposed to digestive enzymes such as pepsin and the protein’s status as a potential allergen, but over time the conclusions of these earlier studies were not confirmed. Nonetheless, many regulatory authorities, including the European Food Safety Authority (EFSA), continue to require digestibility analyses as a component of GM crop risk assessments. Moreover, EFSA has recently investigated the use of mass spectrometry (MS), to make digestion assays more predictive of allergy risk, because it can detect and identify small undigested peptides. However, the utility of MS is questionable in this context, since known allergenic peptides are unlikely to exist in protein candidates intended for commercial development. These protein candidates are pre-screened by the same bioinformatics processes that are normally used to identify MS targets. Therefore, MS is not a standalone allergen identification method and also cannot be used to predict previously unknown allergenic epitopes. Thus, the suggested application of MS for analysis of digesta does not improve the poor predictive power of digestion assays in identifying allergenic risk.

## Introduction

### Background

The safety of genetically modified crops is evaluated in many ways, including testing and characterizing the molecular and biochemical outcomes of plant transformation. Primary concerns center around ensuring that negligible consumer risk, including protein toxicity or allergenicity, are presented by newly expressed proteins in GM crops. Although toxicity in consumers can be predicted using the results of a number of in vitro and in vivo test systems, no such systems are completely predictive of allergenic risk. Therefore, a weight of evidence is required to support the evaluation of allergenic risk.

Scientific opinions regarding the types of data needed for the assessment of potential allergenicity are continuously evolving. In the 1970s and 80s, several investigators became interested in defining the immunologic properties of allergenic proteins, with a focus on the potential correlation between protein stability to digestion and allergenicity (e.g., Haddad et al., 1979; Schwartz et al., 1980; Taylor, 1986; Taylor et al., 1987). Testing protein stability following exposure to simulated gastric fluid (SGF) and simulated intestinal fluid (SIF) was first incorporated into a safety assessment for a genetically engineered plant by Fuchs et al. (1993). Around the same time, a paper supporting a relationship between the stability of proteins following in vitro exposure to SGF and allergenic status was published (Astwood et al., 1996). Based largely on the Fuchs and Astwood publications (Astwood et al., 1996; Fuchs et al., 1993), results of in vitro digestibility assays were incorporated into the allergenic risk assessment of newly expressed proteins in GM crops as advocated by Metcalf et al. (1996), and study requirements were codified into regulatory guidance (FAO/WHO, 2001; CODEX, 2009). The support for resistance to in vitro digestion being correlated with allergenicity was then reinforced by the observation that individuals taking antacids have an increased risk of food allergy, ostensibly due to reduced gastric digestion of proteins at higher pH levels (Untersmayr et al., 2003).

**Fig. 1:**
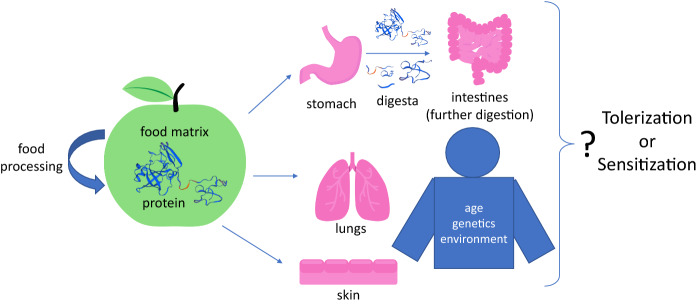
Do we know how increased or decreased exposure to a protein in the gut affects tolerization vs. sensitization? With the knowledge that sensitization to classic food allergens can occur through inhalation and dermal exposure to food proteins, and that exposure to allergenic foods at a young age reduces the frequency of allergy later in life, the lack of correlation between the digestive stability and allergenicity of proteins is now unsurprising. The effects of the food matrix and food processing, and the age, genetics, and environment (including microbiome composition) of individuals, further complicate predictions. With this backdrop, it is unclear how identifying small peptides in digesta will meaningfully inform the allergenicity risk assessment for newly expressed proteins in genetically engineered crops

Since that time, evidence has accumulated that the digestive stability of proteins is actually a very unreliable predictor of the allergenic status of proteins (Fu et al., 2002; Torcello-Gomez et al., 2020; Herman et al., 2007; Akkerdaas et al., 2018). However, the intuitive appeal of this relationship, based on the model that food-allergy sensitization and elicitation occur primarily in the gut, has persisted (Herman et al., 2020). Recent developments indicating that sensitization and elicitation by proteins traditionally thought of as food allergens can also occur both through dermal and respiratory routes of exposure (Turcanu et al., 2017; Inomata et al., 2015; Herman and Ladics, 2018) (Fig. 1), and that antacid users also have higher rates of dermal (e.g., eczema) and respiratory (e.g., asthma) immune disorders, have challenged this belief (Robinson and Camargo Jr, 2018). It is now becoming apparent that the effect of antacids on the microbiome (a known modulator of immune responses), rather than impaired gastric digestion, is the likely mechanism by which allergy is increased (Pascal et al., 2018), similar to increased respiratory, dermal, and food allergy associated with antibiotic use (Riiser, 2015; Hirsch et al., 2017; Herman, 2020). Consequently, it is now beginning to be more widely recognized that in vitro digestibility, while potentially pertinent to exposure in the gut, is not of value in distinguishing allergens from non-allergens (Bøgh and Madsen, 2016; Herman et al., 2020; Verhoeckx et al., 2019).

**Fig. 2:**
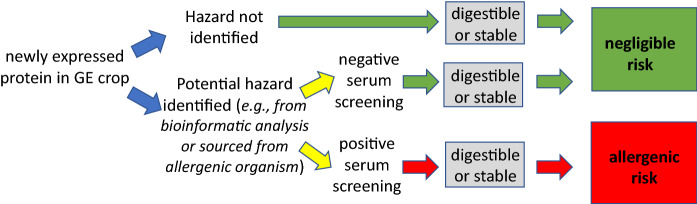
Allergy elicitation-assessment decision flow showing digestibility as a non-decision factor. Note that negative clinical testing could mitigate positive serum screening results

Exposure in the gut of sensitized individuals to the offending food allergens can be reduced through gastric and intestinal digestion subsequently reducing allergenic symptoms (elicitation). However, if a newly expressed protein in a GM crop is found to present a potential allergenic hazard based on bioinformatic criteria (amino acid sequence similarity to known allergens) or was sourced from an allergenic organism, and this potential allergenic hazard cannot be dismissed based on available evidence, then screening against specific IgE antibodies in serum from individuals sensitized to the relevant known allergen is conducted. If such testing reveals lack of cross-reactive binding, then it can be concluded that the newly expressed protein is not a cross-reactive risk. If cross reactivity is observed, then development of the crop expressing that protein will be discontinued unless clinical studies show lack of elicitation of allergenic symptoms (Herman and Ladics, 2018). Since some allergens are digested very rapidly, observing rapid digestion of the newly expressed protein would not preclude it being an allergenic risk, and in the absence of observed cross reactivity, stability would not indicate risk. Thus, results of digestion studies, while pertinent to exposure in the gut, are not a reliable source of information to support the allergenicity risk assessment of newly expressed proteins in GM crops (Fig. 2).

Regardless of the lack of scientific evidence, most risk assessment bodies request that in vitro digestive results be provided as a part of an allergenicity assessment of newly expressed proteins in GM crops. Historically, protein digestibility has been assessed using sodium dodecyl sulfate polyacrylamide gel electrophoresis (SDS PAGE). Digestion is evaluated based on the rate of disappearance of the band representing the intact protein, and/or the appearance of lower molecular weight bands over time. The method has been criticized because fragments < 3 kDa molecular weight are not easily visualized. Recently, the European Food Safety Authority GMO Panel explored the implementation of tandem mass spectrometry (MS) in conjunction with SDS PAGE to more comprehensively map protein/peptide digesta and identify small stable digestion fragments (EFSA Panel on Genetically Modified Organisms et al., 2017).

## Mass spectrometric detection of small peptides

Technical advancements in the application of mass spectrometry (MS) to identify small peptides in complex mixtures have been used to characterize the processing of known allergens and non-allergens exposed to digestive enzymes (Mackie et al., 2019; Korte et al., 2017; EFSA Panel on Genetically Modified Organisms et al., 2017; Wang et al., 2020; Di Stasio et al., 2020). No pattern of peptide fragmentation was found to be associated with the allergenic status of proteins (Torcello-Gómez et al., 2020; Wang et al., 2020). MS can be useful in the identification of epitopes contained in small peptides within the digestive residues of known allergens, which can then be further investigated using IgE antibodies from sensitized individuals, but such analyses are incapable of identifying unknown allergenic epitopes in proteins not known to cause allergy. In the case of newly expressed proteins in GM crops, bioinformatic analyses are used to ensure the absence of peptide sequences associated with allergy in trait proteins, especially peptides that might contain cross-reactive IgE epitopes. Indeed, in most cases, bioinformatic identification of shared significant amino acid similarity with known allergens precludes development of such transgenic events (Ladics et al., 2011). Thus, it is difficult to formulate a scenario where MS analyses for small peptides in the digesta from newly expressed proteins in GE crops would inform an allergenicity risk assessment.

## 2017 cost action ImpARAS and cost acton INFOGEST workshop

A COST Action is a topic-specific scientific research network funded by The European Cooperation in Science and Technology (www.cost.eu/cost-actions/what-are-cost-actions/#) using EU taxpayer funds. Two COST Actions (ImpARAS, for “Improving Allergy Risk Assessment Strategy for New Food Proteins”, at www.imparas.eu and INFOGEST, for “Improving Health Properties of Foods by Sharing our Knowledge on the Digestive Process” at www.cost-INFOGEST.eu\) recently convened a workshop on the relevance of a digestibility evaluation in the allergenicity risk assessment of novel proteins (Verhoeckx et al., 2019). In the conclusions of the workshop, it was stated:

Moreover, there is no rationale on which to base a clear readout that is predictive for allergenicity exclusively and the exact route of exposure and mechanisms behind food sensitization and food allergy are not fully understood yet. Therefore, we suggest omitting the digestion test from the allergenicity assessment strategy for now and put an effort into filling the knowledge gaps. Finally, any digestion assay developed to support the allergenicity assessment of novel dietary proteins should be validated and produce results that can distinguish known allergens from non-allergens with a reasonable level of selectivity.

Fundamental data gaps identified by this group include the role of elevated pH in digestion and the influence of meal composition on pH levels, the role of microbiota in food allergy, the mechanisms by which food allergens migrate and interact with the immune system, the mechanism of sensitization, the effect of peptide size on sensitization and elicitation, a better understanding of appropriate parameters to measure and methods to measure them, as well as gaps in several others areas (Verhoeckx et al., 2019).

## Conclusions

This synopsis concurs with the conclusions of the 2017 COST Action ImpARAS and COST Acton INFOGEST Workshop that the current use of digestion results to inform the allergenicity risk assessment for newly expressed proteins in GM crops is of low value and new developments have not improved the ability of digestion assays to “…*distinguish known allergens from non-allergens with a reasonable level of selectivity.*” Furthermore, this synopsis agrees that “…*any digestion assay developed to support the allergenicity assessment of novel dietary proteins should be validated…*” (Verhoeckx et al., 2019).

Increasingly, the evidence surrounding the allergenicity risk assessment for GM crops indicates that digestion assays are of little value in the context of protein allergenicity. Recent advancements in using MS to identify small peptides in digesta has not improved the value of digestion assays for the assessment of allergy risk (Mackie et al., 2019; Wang et al., 2020, 2021). Therefore, the weight of the current evidence surrounding the allergenicity risk assessment for GM crops suggests that digestion assays should not be considered unless a validated assay with proven criteria is developed that can distinguish allergens from non-allergens with some reasonable level of reliability (Verhoeckx et al., 2019; Herman et al., 2020; Bøgh and Madsen, 2016). The ability to use MS to detect small peptides in digesta does not improve the determination of allergenic potential, and therefore, does not change this conclusion. Thus, the application of MS to detect small peptides in digests is not useful in the assessment of the allergenic assessment of newly expressed proteins in GE crops.
